# Analysis of the individual profile of children in Autism Spectrum Disorder (ASD) and therapeutic strategies in the DIR/Floortime model

**DOI:** 10.1192/j.eurpsy.2023.1557

**Published:** 2023-07-19

**Authors:** P. M. Pacheco, P. Piacentini, M. D. S. Pacheco, D. R. Molini-Avejonas

**Affiliations:** 1 Speech Language Pathology, University of São Paulo, são Paulo; 2 Special Education, CDI/ICDL, Recife; 3 Morphology, UFES, Vitoria, Brazil

## Abstract

**Introduction:**

Children with Autism Spectrum Disorder (ASD) have a qualitative deficit in social interaction, engagement, and behavior. The DIR/Floortime model is one of the ways of intervention and is based on the child’s Functional Development, Individual differences, and Relationships. It aims to build the foundation for the social, emotional, and intellectual skills of children, instead of having the focus only on isolated behaviors. The model was developed by Stanley Greenspan and Serena Wieder in the United States and is the result of many years of observations and studies on child development since the 1950s. In the 1980s, they unified knowledge from several related studies on child development and mental health and recognized the importance of relationships and affection for learning. One of the considerations of the DIR/Floortime model on children with autism is the individual profile, that is, their individual differences (the I of the DIR). Each child has a unique way of perceiving the world (sight, sounds, touch) and responding to it. They may have difficulties in processing or responding to sensory information. Their individual differences need to be well known so that we can draw up a therapeutic plan to obtain the best developmental evolution.

**Objectives:**

Recognize and analyze the individual differences of each child, so that the appropriate therapeutic plan can be traced for the development of their potential.

**Methods:**

Participated in the study 63 children with ASD, 12 girls (19%) and 51 boys (81%). Global Development Assessment questionnaires were used, based on the FEDC and the FEAS scale of the DIR/Floortime.

**Results:**

All 63 children presented sensory alterations such as proprioceptive, visual, and vestibular search or hyper-reactivity, directly impacting abilities such as visuospatial processing and motor planning. In addition, 85% of children have low body tone. Regarding the sensory need for visual search, presented by 86% of the children, as well as the vestibular (90%), a recommended therapeutic strategy is mapping the place, with fewer objects. The therapist needs to be in a fixed position and maintain a pleasant, lower tone of voice. The routine framework for motor and action planning, anticipating, and giving the necessary waiting time for the child to get organized. Motor circuits can also help to work with the tone, as well as with praxis.

**Conclusions:**

The DIR/Floortime model aims to make the child develop the ability to interact meaningfully and connect with the outside world. The individual differences of the child need to be known so that this work can take place effectively and so that the therapist can better organize the therapy, providing the best development for the child.

**Disclosure of Interest:**

None Declared

